# Annotations

**Published:** 1904-07-09

**Authors:** 


### ANNOTATIONS.

### A Commercial Standard.

THE controversy in respect to the question whether the British Pharmacopœia is the one and only standard for all articles named and defined in its pages continues to exercise the magisterial bench. No one denies that it is such a standard in the interpretation and dispensing of physicians' prescriptions. The difference of opinion arises in relation to the retail sale of articles which have both a medicinal and a popular or industrial use. The latest illustration is provided by a case in which a retailer was summoned under the Sale of Food and Drugs Act for supplying ammonium carbonate which was not of the strength of the pharmacopœial standard. It was admitted that the substance supplied did not satisfy the official tests, but counsel for the defence contended that it was not sold as a drug, but for commercial use, in accordance with a popular demand and custom. Several witnesses of long experience in the drug trade gave evidence in favour of this view, and stated that the practice of pharmacists was to keep the official ammonium carbonate for dispensing purposes and the commercial variety to sell to the public, who used it as a cleansing agent. The magistrates held that, in the case submitted, there had been no sale of a drug, and that therefore the ammonium carbonate was not liable to the pharmacopœial tests. This, it seems to us, is a reasonable and common-sense view to adopt. That the official standard should be rigidly upheld in the case of medicines is only right and proper. But to attempt to make this standard govern commercial transactions, which are a popular convenience, and which involve no prejudice to the purchaser, is calculated to do harm rather than good. It is apt to bring the law into contempt and also to increase the price of a number of articles for which there is a popular demand, and which, in their ordinary commercial forms, fulfil numerous popular and domestic purposes.

### Cheap Alcohol.

ALCOHOL as a beverage is the centre of a number of acute, social, and political controversies with which in these columns we have no direct concern. But there are many other purposes for which alcohol is used, and certain of these have considerable economic and even some degree of scientific importance. It is employed in many manufacturing and industrial occupations, and more recently has become of interest to the automobilists. Representatives of the chemical industry and some of the Chambers of Commerce have more than once pointed out that various trades are handicapped by the fact that alcohol used for commercial purposes is, equally with that used as a beverage, subject to a heavy excise duty. They have now been joined by the Automobile Club, and there is reason to believe that, under the joint pressure so exerted, the Chancellor of the Exchequer may be induced to order an inquiry into the whole subject. The difficulty of course is to establish conditions which will prevent abuse and will also protect the public revenue. It is obvious that if untaxed alcohol is to be supplied care must be taken to prevent its use for other than manufacturing and similar purposes. Experience has shown that alcohol mixed with a certain percentage of wood spirit and sold as methylated spirit, even though the sale is safeguarded by careful restrictions, may find its way to the thirsty throat. Hence it was necessary some few years ago to qualify the alcohol still further by the addition of a percentage of mineral spirit. This in its turn has produced some inconvenience, especially in the use of methylated spirit for certain scientific and medicinal uses. A non-mineralised methylated spirit may, it is true, in certain circumstances be obtained, but the regulations which govern such a transaction are complicated and somewhat arbitrary. The whole question of duty-free alcohol is therefore one eminently worthy of careful study, and it is to be hoped that the authorities will grant the inquiry which has been suggested. It ought not to be beyond the wit of man to devise a scheme which will relieve the manufacturer of an unnecessary tax, and will at the same time avoid the chance of illegitimate drinking and protect the public purse.

### Cold-storage in Hospitals.

ICE-MAKING and cold storage apparatus at the present day constitute a large industry with important commercial relationships. The industry possesses a monthly organ from which we learn of an interesting installation recently carried out at the Middlesex Hospital. This includes a special refrigerating plant, distilled water ice-making appliances, insulated cold chambers for provisions, etc., a refrigerated mortuary, and a cold chamber for preserving pathological specimens. The system of refrigerating machinery is that of Messrs. Ransomes and Rapier, Limited, and one of its special advantages is that there are no engines, motors, compression pumps, or foundations required, so that all noise and vibration being absent the apparatus may be erected in any part of the hospital. The principle is that of ammonia absorption, and the refrigerating machine is of a special pattern approved by the Board of Trade for use in passenger steamers. It is claimed for this that it combines the safety of cold air and carbonic acid machines with the greater economy and efficiency of ammonia machines. In practice at the hospital it is found that working the machine a few hours a day is sufficient to secure in the various chambers a steady temperature of 20° F., a constant circulation of cold brine being maintained in the intervals during which the machine is not in operation. The tests in reference to efficiency therefore appear to be satisfactorily answered. The claim for economy seems to be met with equal assurance. The ice-making appliances are able to make a ton of distilled water ice per day, and this it is found costs only one-third of the price formerly paid. The distilled water is obtained free of cost from the condensed steam produced in working the refrigerating machine, and as no lubricating oils are required the condensed steam is singularly free from contamination. A further point of importance is that the apparatus is of such simplicity that it neither requires much nor skilled attention. Altogether the arrangement may be recognised as a decided advance in a department which concerns both the economic and scientific sides of hospital management.

